# Beyond m^6^A: The Expanding Landscape of mRNA Modifications in Renal Cell Carcinoma

**DOI:** 10.3390/biomedicines14091962

**Published:** 2026-08-31

**Authors:** Zongchen Hou, Diaoyi Tan, Zhiyong Xiong, Daojia Miao

**Affiliations:** 1 Department of Urology, Union Hospital, Tongji Medical College, Huazhong University of Science and Technology, Wuhan 430022, China; zongchenhou@163.com (Z.H.); d202281965@hust.edu.cn (D.T.); 2 Institute of Urology, Union Hospital, Tongji Medical College, Huazhong University of Science and Technology, Wuhan 430022, China

**Keywords:** renal cell carcinoma, epitranscriptomics, mRNA modification, therapeutic resistance

## Abstract

Renal cell carcinoma (RCC) comprises molecularly diverse tumor subtypes. This diversity is reflected in distinct and adaptable gene-expression programs that enable tumor cells to reprogram metabolism and survive therapeutic pressure. By regulating RNA processing, export, stability, decay, and translation, messenger RNA (mRNA) modifications may help shape these programs. Examining mRNA modifications is therefore important for understanding metabolic adaptation and treatment resistance in RCC. This narrative review focuses on RCC studies of N6-methyladenosine (m^6^A), 5-methylcytosine (m^5^C), N7-methylguanosine (m^7^G), and N4-acetylcytidine (ac^4^C). We focus on protein-coding transcripts and mRNA-centered mechanisms. Transcript-level studies are most extensive for m^6^A, while selected m^5^C and ac^4^C studies have identified defined regulator–mRNA axes. By comparison, RCC data for specific internal m^7^G sites in mRNA remain largely indirect. Many reported links with immune features or treatment response are based on retrospective analyses rather than treatment-specific clinical validation. Different modification pathways converge on PI3K/AKT, Hippo/YAP, metabolism, and treatment resistance, although direct molecular crosstalk has not been shown in RCC. No RNA-modification biomarker or targeted agent is used in routine RCC care. Further progress will require orthogonal site validation, broader coverage of RCC subtypes, and prospective studies conducted within contemporary treatment settings.

## 1. Introduction

Renal cell carcinoma (RCC) is a clinically and molecularly diverse group of tumors, dominated by clear cell RCC (ccRCC). Genomic alterations, metabolic reprogramming, the tumor microenvironment, and acquired resistance all contribute to disease progression and treatment outcomes [1,2,3]. Messenger RNA (mRNA) modifications add another regulatory layer by affecting RNA processing, nuclear export, stability, decay, and translation [4,5,6,7]. Their effects depend on the transcript, the regulatory protein, and the cellular setting. In RCC models, these processes have been linked to tumor growth, metabolic adaptation, immune phenotypes, and drug resistance.

Recent reviews have discussed RNA-modifying proteins in RCC and RNA methylation across urological cancers [8,9]. The present review takes a narrower, transcript-centered approach. It focuses on protein-coding mRNAs and considers four modifications: m^6^A, m^5^C, m^7^G, and ac^4^C. We retained m^7^G as a separate section because several RCC studies have used m^7^G-related regulator sets, although direct evidence for internal m^7^G sites in RCC mRNA remains limited. m^1^A is discussed as an understudied area because the available RCC studies are limited to regulator-expression and retrospective computational analyses, without transcript-specific modification evidence. Studies are discussed according to what was measured, from direct transcript-level assays to regulator studies and retrospective signatures. This approach also separates shared pathway involvement from direct molecular crosstalk. Clinical relevance is considered in the context of current immune checkpoint inhibitor (ICI)-based combinations.

We therefore summarize the molecular machinery of these modifications and review their reported roles in RCC. The discussion is organized around tumor progression, metabolism, immune regulation, and treatment resistance. We then compare pathways shared across modification classes and assess the maturity of proposed biomarkers and therapeutic targets. The aim is to provide a practical account of what is supported, what remains uncertain, and which questions are most relevant for future RCC research.

## 2. Review Methodology

### 2.1. Literature Search and Study Selection

This structured narrative review focuses on mRNA modifications in RCC. PubMed was searched on 14 August 2026 for records published from 14 August 2021 to 14 August 2026. Earlier studies already cited in the manuscript were retained when they provided foundational mRNA biology, established detection methods, or irreplaceable RCC data.

All disease and modification terms were searched in the Title/Abstract field. The common disease block comprised renal cell carcinoma, renal carcinoma, renal cancer, kidney cancer, clear cell renal cell carcinoma, ccRCC, KIRC, papillary renal cell carcinoma, pRCC, KIRP, chromophobe renal cell carcinoma, chRCC, KICH, translocation renal cell carcinoma, tRCC, and NONO-TFE3. This block was combined separately with m^6^A-related terms (m^6^A, N6-methyladenosine, METTL3, METTL14, METTL16, WTAP, KIAA1429, VIRMA, RBM15, RBM15B, ZC3H13, FTO, ALKBH5, YTHDF*, YTHDC*, and IGF2BP*), m^5^C-related terms (m^5^C, 5-methylcytosine, RNA 5-methylcytosine, NOP2, NSUN2, NSUN5, NSUN6, TRDMT1, ALYREF, and YBX1), m^7^G-related terms (m^7^G, 7-methylguanosine, N7-methylguanosine, internal m^7^G, METTL1, WDR4, RNMT, and BUD23), or ac^4^C-related terms (ac^4^C, N4-acetylcytidine, RNA cytidine acetylation, NAT10, and THUMPD1).

Eligible records were English-language original studies of RCC or a defined RCC subtype. Studies had to examine one of the four modifications or its regulatory machinery and identify a protein-coding mRNA substrate or an mRNA-centered mechanism. Non-RCC reports were retained only for essential biochemical or methodological background. We excluded reviews, conference abstracts, corrections, retracted reports, records with an indeterminate RNA species, and studies confined to lncRNA, circRNA, miRNA, tRNA, or rRNA substrates. Purely computational models were excluded from the updated mechanistic set unless they added a distinct biological or clinical question.

### 2.2. Study Categorization and Interpretation

RCC studies were categorized according to the measurements supporting their main conclusion. C1 denotes direct measurement of a modification in RCC mRNA and was subdivided by analytical resolution. C1a includes site-directed manipulation, site-specific detection, or orthogonally supported modification-dependent evidence. C1b includes candidate-site mutation, reporter assays, or targeted-site experiments without chemical single-nucleotide confirmation. C1c includes transcript-specific enrichment or peak-level detection without site validation. C1d includes global, method-dependent, non-orthogonally validated, or abstract-reported mapping evidence. C2 includes regulator–target mRNA mechanisms supported by perturbation, binding, stability, translation, or rescue experiments without direct measurement of the modification on that transcript. C3 covers regulator expression or function when a modified target mRNA was not verified. C4 includes retrospective molecular subtyping, prognostic scores, immune associations, and drug-sensitivity inference. These categories describe analytical resolution, study type, and modification specificity; they are not risk-of-bias or clinical-maturity scales.

Each original RCC study was normally treated as one unit. Separate conclusions from the same paper could be listed independently when their experimental support differed. For boundary studies involving a noncoding upstream or interacting transcript, categorization followed the protein-coding mRNA substrate. Non-RCC biochemical and methodological references were used as background. Conclusions were limited to the resolution of the assay and the design of the study. Clinical prediction or treatment utility required independent clinical validation.

## 3. Types and Regulatory Mechanisms of Major mRNA Modifications

Epitranscriptomic regulation refers to covalent RNA modifications that influence RNA processing and function during or after transcription. This review considers four modifications implicated in RCC: N6-methyladenosine (m^6^A), 5-methylcytosine (m^5^C), N7-methylguanosine (m^7^G), and N4-acetylcytidine (ac^4^C). Their regulatory systems are not equally understood. m^6^A has well-characterized writers, erasers, and readers, whereas internal m^7^G and mRNA ac^4^C remain less clearly mapped [10,11].

### 3.1. m^6^A Modification

m^6^A is a common internal modification of eukaryotic mRNA. It is enriched near stop codons and in 3′ untranslated regions, often within the RRACH sequence context [12,13]. Deposition can occur during transcription, after which m^6^A can influence pre-mRNA processing, nuclear export, stability, decay, and translation [14,15].

The core methyltransferase complex contains METTL3 and METTL14. METTL3 provides the main catalytic activity, while METTL14 stabilizes the complex and assists RNA recognition. WTAP, VIRMA/KIAA1429, RBM15/RBM15B, and ZC3H13 help localize the complex and select substrates [15,16,17]. FTO and ALKBH5 can remove methyl marks from RNA, although their preferred substrates and biological effects vary with localization and cellular context [15,18].

Major m^6^A-associated proteins include YTHDC1, YTHDC2, YTH domain-containing family proteins 1–3 (YTHDF1–3), and the reported noncanonical readers IGF2BP1–3 [15,19,20]. YTHDC1 acts mainly in the nucleus, whereas YTHDF proteins are predominantly cytoplasmic. YTHDC2 contains both RNA helicase and YTH domains. IGF2BP proteins have been linked to increased stability or translation of selected transcripts. Reader effects remain transcript- and context-dependent, and individual proteins cannot be assigned a single universal effect on mRNA fate [21,22].

### 3.2. m^5^C Modification

m^5^C is produced by methylation of cytidine at carbon 5. It is abundant in tRNA and rRNA but has also been reported in mRNA. Estimates of the abundance of m^5^C in mRNA and its distribution vary between methods because RNA structure and incomplete conversion can affect bisulfite-based mapping [10,23]. No universal sequence motif has been established for mRNA m^5^C.

NSUN2 and NSUN6 are the principal enzymes associated with m^5^C deposition on mammalian mRNA, although their substrates differ [24,25]. TET-family enzymes can oxidize RNA m^5^C, but they are better described as oxidation-associated enzymes than as universal erasers of m^5^C in mRNA [26,27]. ALYREF has been linked to nuclear export of m^5^C-containing transcripts, while YBX1 can stabilize selected m^5^C-containing mRNAs [25,28].

Work outside RCC has identified high-stoichiometry, site-specific NSUN6-mediated m^5^C on a group of transcripts [29]. This establishes the capacity of NSUN6 to modify mRNA but does not identify an RCC-specific substrate or phenotype.

### 3.3. m^7^G Modification

m^7^G occurs in two distinct settings. The established 5′ mRNA cap is produced by guanylylation and methylation of cap guanosine by the RNMT-RAM complex. Recognition by cap-binding proteins such as eIF4E supports mRNA stability, export, and translation initiation [30,31,32].

Internal m^7^G has also been reported in mammalian mRNA using antibody- and sequencing-based approaches [33,34]. METTL1-WDR4 is best established as a tRNA m^7^G methyltransferase and is distinct from RNMT-dependent capping. Proposed internal mRNA sites remain sensitive to the detection method and experimental setting [34,35,36]. No accepted eraser, reader, or universal motif has been identified for internal m^7^G in mRNA.

Candidate internal m^7^G can be studied by transcriptome-wide enrichment, differential enzymatic digestion, and mass spectrometry [37,38]. These methods support discovery, but any specific RCC site still requires orthogonal confirmation.

### 3.4. ac^4^C Modification

ac^4^C is formed when an acetyl group is added to the N4 position of cytidine. NAT10 is the principal established ac^4^C acetyltransferase in human cells. Mammalian studies have linked NAT10-dependent ac4C in mRNA to RNA stability and translational efficiency [39,40]. However, estimates of its distribution and stoichiometry remain method-sensitive, and individual sites require orthogonal validation [10,41].

THUMPD1 acts as an adaptor for NAT10-dependent acetylation of selected tRNAs, but a general adaptor role in mRNA ac^4^C has not been shown [42]. No mRNA-specific ac^4^C eraser, reader, or universal sequence motif is currently accepted [10,43].

**Figure 1:**
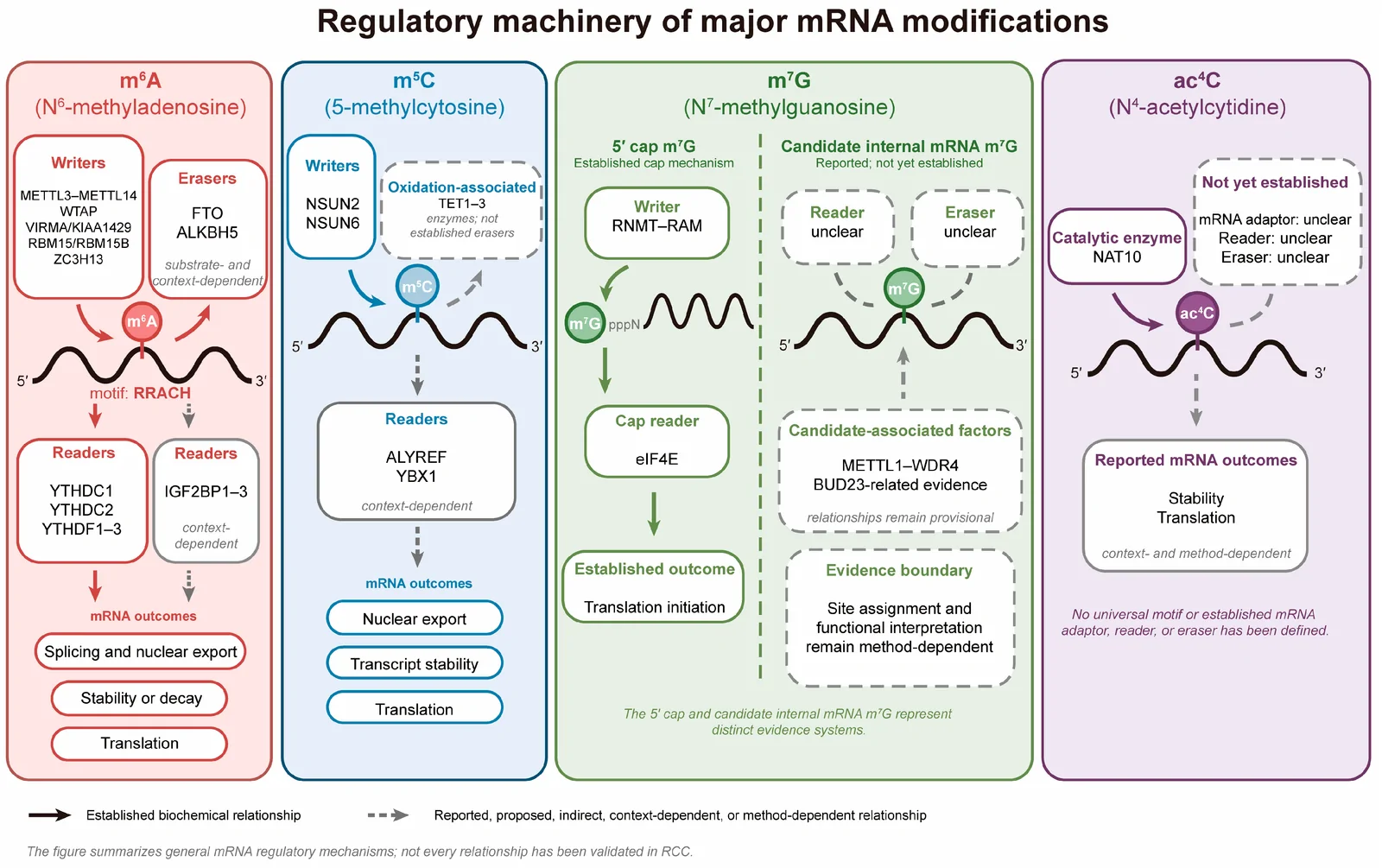
Regulatory machinery of major mRNA modifications. Colored solid boxes indicate established biochemical components, gray solid boxes indicate reported or context-dependent evidence, and gray dashed boxes indicate roles or relationships that remain provisional or unclear. Solid arrows represent established biochemical relationships, whereas gray dashed arrows represent reported, proposed, indirect, context-dependent, or method-dependent relationships. The 5′ m7G cap is shown separately from candidate internal m7G in mRNA. This figure summarizes general mRNA regulatory mechanisms; not all depicted relationships have been validated in RCC.

Figure 1 outlines the general regulatory machinery and distinguishes established relationships from proposed or method-dependent links.

## 4. m^6^A Modification in RCC

### 4.1. m^6^A Regulators and mRNA Targets in Tumor Progression

The effects of m^6^A in RCC depend on the regulator and the target transcript. In ccRCC, METTL14 reduced ITGB4 mRNA stability and limited epithelial–mesenchymal transition and PI3K/AKT signaling [44]. VHL promoted formation of the METTL3/METTL14 complex and regulated PIK3R3 mRNA stability, linking VHL loss to m^6^A-dependent PI3K/AKT activity [45]. Conversely, KIAA1429/VIRMA stabilized MYC mRNA and promoted ccRCC growth and invasion [46]. METTL3 also enhanced ZNF677 mRNA stability and translation through IGF2BP2 and YTHDF1. ZNF677 subsequently suppressed RCC progression by repressing CDKN3 [47]. These studies illustrate why the direction of an m^6^A effect cannot be inferred without knowing the target transcript.

Recent studies have identified additional writer- and eraser-associated targets. METTL16 increased m^6^A enrichment of KLK4 mRNA and supported IGF2BP1-dependent KLK4 expression [48]. KIAA1429 was also associated with increased m^6^A enrichment of TMSB10 mRNA and tumor-promoting effects [49]. The available abstract did not describe the site resolution in sufficient detail. METTL14 stabilized ZFP14 mRNA through IGF2BP2, and ZFP14 promoted STAT3 ubiquitination [50]. Other reports linked FTO to POLQ mRNA and genome stability [51], prolonged hypoxia to a METTL3-PLOD2 axis [52], and METTL3/IGF2BP1 to ZHX2 mRNA stability [53]. These studies did not provide nucleotide-resolved chemical confirmation of the proposed sites.

Reader proteins add further context. Lactylation of YTHDC1 at K82 altered its phase separation and stabilized BCL2 and E2F2 mRNAs [54]. This is a protein post-translational modification that changes reader activity, not direct crosstalk between two RNA modifications. IGF2BP2 recognition of m^6^A-modified TGF-β-SMAD transcripts reduced cell growth but increased migration and metastasis, with the strongest site-level data reported for SMAD4 [55]. IGF2BP2 also stabilized Netrin-4 mRNA and limited ccRCC migration and invasion, although that study lacked in vivo validation [56].

The tumor stroma may also influence m^6^A regulation in pRCC. Cancer-associated fibroblast-derived lactate increased histone lactylation and RBM15B expression. RBM15B then increased m^6^A enrichment and stability of ANLN mRNA in paired tumors and fibroblast-based models [57]. This pathway connects stromal metabolism with m^6^A regulation without demonstrating crosstalk between RNA modifications.

### 4.2. Metabolic Reprogramming and Cellular Stress

Several m^6^A-regulated transcripts influence RCC metabolism. FTO reduced OGDHL mRNA stability, allowing TFAP2A-dependent FASN transcription, lipid accumulation, and ERK activation [58]. METTL3-mediated m^6^A destabilized DBT mRNA, disrupted ANXA2/YAP-regulated Hippo signaling, and favored lipid accumulation [59]. L-2-hydroxyglutarate inhibited ALKBH5 and FTO, increased mRNA m^6^A, and suppressed serine-biosynthesis genes such as PSAT1 [60]. FTO also regulated autophagic flux through an m^6^A-IGF2BP2-SIK2 pathway [61]. Together, these studies connect m^6^A with lipid metabolism, serine dependence, Hippo/YAP signaling, and autophagy.

Newer reports extend these observations to lipogenesis, glutamine use, and oncometabolite signaling. CDK13 phosphorylated METTL16 and increased m^6^A enrichment of ACLY mRNA, which was stabilized through YTHDC2 [62]. METTL14 and ACSM3 were linked to reduced lipid accumulation through GATA5/HMGCS2 and mTORC1 signaling [63]. However, that study did not directly measure m^6^A on ACSM3 mRNA. FTO regulation of SLC1A5 supported glutamine metabolism and growth in VHL-deficient ccRCC, although the modification-specific link relied on earlier work [64]. In FH-deficient cells, fumarate accumulation increased global and peak-level mRNA m^6^A. The study did not show that this increase caused global changes in mRNA stability or translation [65].

Ferroptosis and stress tolerance provide two additional settings. Loganin destabilized IGF2BP2, reduced its binding to m^6^A-containing HMOX1 mRNA, and increased ferroptotic injury in cell and xenograft models [66]. METTL3/IGF2BP1-dependent stabilization of MANF mRNA promoted endoplasmic-reticulum stress tolerance in RCC models [67]. Both observations remain preclinical, and the MANF pathway has not been linked to clinical treatment resistance.

### 4.3. Immune-Related Mechanisms and TKI Resistance

m^6^A studies of treatment response include direct resistance mechanisms, experimental compounds, and retrospective immune analyses. YTHDC1 reduced the stability of m^6^A-marked ANXA1 mRNA and altered MAPK signaling and sunitinib sensitivity [68]. METTL3 increased m^6^A enrichment and stability of TRIM37 mRNA, activating the TRIM37-SMARCC2-Wnt pathway [69]. Neither study mapped the modified nucleotide directly. They nevertheless identify testable mechanisms of TKI resistance.

Experimental compounds have also been studied. Erianin increased m^6^A signals on ALOX12 and TP53 mRNAs and induced ferroptosis in renal cancer stem-cell models [70]. The FTO inhibitor FB23-2 reduced tumor growth in ccRCC patient-derived xenograft models [61]. These findings support preclinical investigation, not clinical efficacy. A separate regulator-derived m^6^Ascore was associated with survival in one retrospective anti-PD-1 cohort [71]. Its treatment-predictive value has not been tested prospectively.

Immune-related mechanisms extend beyond retrospective scores. Loss of ZC3H13 reduced m^6^A enrichment of UNC5CL mRNA and promoted myeloid-derived suppressor cell recruitment through YTHDC1 and NF-κB/CSF2 signaling [72]. A STAT1/METTL3-associated IL15RA axis promoted metastasis through ZEB1/NF-κB signaling, although IL15RA modification was not measured directly [73]. YTHDF3 phase separation promoted HSPA13 mRNA decay and immune-evasion phenotypes [74]. HSPA13 modification was inferred, and the pathway has not been tested as a predictor of ICI response. A separate ccRCC study found that METTL3-dependent m6A enrichment of NCAPH mRNA supported YTHDC1-associated nuclear export and IGF2BP3-associated mRNA stability. NCAPH increased PD-L1 expression and reduced anti-PD-1 activity in an immunocompetent mouse model, although its treatment-predictive value has not been evaluated clinically [75].

### 4.4. Current Evidence and Clinical Context for m^6^A

Additional resistance pathways have been studied in specific drug settings. ZC3H13 stabilized sFRP4 mRNA and reduced Wnt/β-catenin signaling in sunitinib-resistant cells and xenografts [76]. The clinical and animal cohorts were small. YTHDF3 binding to SYNM mRNA contributed to reversible resistance to the experimental PI3K/mTOR inhibitor NVP-BEZ235 [77]. Neither pathway has been evaluated in contemporary ICI–TKI combinations.

m^6^A has the broadest transcript-level mechanistic literature among the four modification classes reviewed here. It links defined mRNAs with tumor progression, metabolism, immune phenotypes, and TKI resistance. Drug studies remain confined to cells, xenografts, or patient-derived xenografts, while immune and prognostic scores remain retrospective. A Nanopore study of one paired ccRCC tumor and adjacent sample illustrates the current discovery potential but also the limitations of very small, algorithm-dependent datasets [78]. No m^6^A biomarker or targeted agent currently informs routine RCC treatment.

The study designs, target transcripts, validation methods, and clinical interpretation of the m^6^A literature are summarized in Table 1.

## 5. m^5^C Modification in RCC

### 5.1. mRNA Targets and Tumor Progression

Four studies have described mRNA-centered m^5^C mechanisms in RCC. PEBP1P2 recruited YBX1/ELAVL1 to m^5^C-containing PEBP1 mRNA, increasing transcript stability and suppressing metastasis [79]. Targeted demethylation and mutation-based assays supported modification dependence. PEBP1P2 itself served as the upstream pseudogene transcript. NOP2 modified APOL1 mRNA, and YBX1 recognized a 3′ UTR m^5^C site to stabilize it [80]. Bisulfite sequencing, MeRIP/RIP, site-mutant reporters, stability assays, and rescue experiments linked this pathway to PI3K/AKT signaling. NSUN5 increased m^5^C enrichment and stability of ENO3 mRNA, promoting glycolysis and tumor growth without nucleotide-resolved validation [81]. ALYREF recognition of m^5^C-modified CCNA1 mRNA increased transcript stability and contributed to pazopanib resistance in cell and xenograft models [82].

### 5.2. m^5^C Regulator Patterns and Immune Associations

Immune-related m^5^C studies have mainly examined regulator expression in retrospective datasets. An analysis of 860 ccRCC samples identified regulator patterns associated with inferred immune infiltration, genetic heterogeneity, and survival [83]. High-score tumors showed an immune-desert-like profile. Because m^5^C was not measured in immune-cell transcripts, the study describes an association rather than a causal change in the tumor microenvironment.

Bulk, single-cell, and spatial transcriptomic analyses also linked m^5^C-regulator expression with immune-cell distributions and produced the M5CRMRGI score [84]. Reported associations with survival, ICI response, and mTOR inhibitor sensitivity came from retrospective datasets and computational drug-response estimates. The score remains exploratory and has not been validated for treatment selection.

### 5.3. Prognostic Studies and Clinical Associations

Several prognostic models used different regulator sets and therefore describe different biological constructs. In pRCC, an ALYREF/DNMT3B/YBX1 signature was developed in TCGA-KIRP and assessed in an ICGC cohort [85]. Its survival, immune, and drug-repurposing findings remained retrospective, and candidate compounds came from connectivity-map analysis. In ccRCC, MARS combined genes assigned to m^6^A, m^5^C, m^1^A, and m^7^G pathways [86]. A separate five-gene score also combined m^5^C and m^6^A regulators [87]. These models were associated with survival and inferred immune features, but they are not m^5^C-specific clinical biomarkers.

Another TCGA/ICGC study grouped ccRCC tumors by m^5^C-regulator expression and derived an m^5^Cscore associated with immune infiltration and drug sensitivity [88]. The analysis did not measure mRNA m^5^C or assess treatment response prospectively.

### 5.4. Current Understanding and Unresolved Questions

RCC data for m^5^C range from direct transcript studies to regulator-expression analyses. The PEBP1, APOL1, ENO3, and CCNA1 pathways provide the clearest mRNA-centered mechanisms [79,80,81,82]. By contrast, compounds proposed for high-risk pRCC were identified computationally and were not tested in patients [85]. Another regulator analysis included limited cell-line work but did not identify a modified target mRNA [89]. These distinctions matter when considering therapeutic relevance.

Recent studies of NSUN proteins add functional information. NSUN6 overexpression was associated with adverse clinicopathological features and promoted ccRCC proliferation, migration, and invasion without identifying a target mRNA [90]. Two NSUN5 studies linked expression or perturbation to ccRCC phenotypes and p53-related signaling, but neither identified a specific NSUN5-modified transcript [91,92]. These studies complement, but do not extend, the direct mRNA mechanisms described above.

YBX1 also promoted LDHA-dependent metabolic reprogramming and NF-κB activation through transcriptional and protein-interaction mechanisms [93]. The study supports a role for YBX1 in ccRCC biology but did not test LDHA m^5^C or modification dependence.

Current m^5^C research in RCC is therefore anchored by a small number of defined mRNA pathways. Immune patterns and prognostic scores remain retrospective, and no m^5^C-derived score or targeted intervention has undergone prospective clinical validation. Replication with orthogonal site-mapping methods and clinically relevant resistance models is needed.

Table 2 compares the direct mRNA mechanisms with regulator-expression studies, retrospective m^5^C analyses, and mixed-modification signatures discussed above.

## 6. m^7^G-Related Studies in RCC

### 6.1. Expression and Functional Roles of m^7^G Regulators

RCC studies of m^7^G have focused on regulator expression and function. METTL1 expression was increased in ccRCC and associated with clinicopathological and survival variables in a TCGA-based study with tissue-microarray validation [94]. Its gene signature and drug-sensitivity estimates were computational, and no modified RCC mRNA was identified. METTL1 or BUD23 knockdown reduced KIRC cell proliferation, colony formation, and migration in another study [95]. Candidate target mRNAs were inferred partly from m^7^G-MeRIP-seq data generated in non-RCC cells. WDR4 knockdown likewise reduced proliferation, migration, and invasion and increased cellular sensitivity to sorafenib and sunitinib in IC50 assays [96]. These studies support regulator-level functions but do not identify specific internal m^7^G sites in mRNA in RCC.

### 6.2. Prognostic and Molecular Classification Studies

Several studies constructed signatures from genes described as m^7^G-related. A five-gene score was developed in TCGA and assessed in E-MTAB-1980 [97]. It was associated with survival and inferred immune and drug-response features but lacked prospective validation. Other reports generated regulator-based clusters, m^7^G scores, or multi-omics signatures from public cohorts [96,98,99]. These models are useful for hypothesis generation, but their clinical value and modification specificity remain uncertain.

### 6.3. Tumor Microenvironment and Treatment Associations

Immune-related conclusions also come mainly from computational analyses. Regulator-based clusters and scores were associated with stromal content, tumor purity, inferred immune-cell distributions, checkpoint-gene expression, and survival [96,98,99]. Most score-based drug-sensitivity estimates were obtained from resources such as GDSC, pRRophetic, and CellMiner rather than prospective RCC treatment cohorts [96,98]. One study additionally included cell-based sorafenib and sunitinib IC50 experiments after WDR4 knockdown [98], but these results remain preclinical. These studies have not shown that internal mRNA m^7^G directly changes the RCC immune microenvironment or predicts response to ICI or TKI treatment.

### 6.4. Current Evidence for Internal mRNA m^7^G in RCC

Current RCC data support associations with m^7^G-related regulators, functional effects of selected proteins, and retrospective gene signatures. Direct nucleotide-resolved validation of an internal m^7^G site in RCC mRNA is still lacking. m^7^G-related gene sets, METTL1-WDR4 or BUD23 function, the established 5′ mRNA cap, and candidate internal m^7^G are distinct concepts. Future studies will need RCC-specific site mapping, orthogonal confirmation, regulator perturbation, and functional rescue before transcript-specific or clinical conclusions can be drawn.

Table 3 summarizes the available regulator-level, functional, and computational m^7^G studies and highlights the absence of directly validated internal mRNA sites in RCC.

## 7. ac^4^C Modification in RCC

### 7.1. NAT10-mRNA Axes in RCC Progression

Two ccRCC studies have described transcript-specific NAT10-mRNA pathways. NAT10-mediated ac^4^C stabilized ANKZF1 mRNA [100]. Modification detection, site mutation, and rescue experiments linked this pathway to YAP1 nuclear localization, pro-lymphangiogenic transcription, tumor growth, and lymphatic metastasis. In a second study, HIF-1α increased NAT10 expression [101]. acRIP-seq, acRIP-qPCR, NAT10-RIP, stability assays, mutant reporters, and rescue experiments supported stabilization of NFE2L3 mRNA. NFE2L3 then promoted LASP1 transcription and AKT/GSK3β/β-catenin signaling. These are the strongest mechanisms involving ac^4^C in mRNA currently reported in RCC.

### 7.2. Functional Studies of NAT10 and THUMPD1

Other studies have examined regulator expression or function without defining an ac^4^C-modified mRNA substrate. In pRCC, NAT10 expression was associated with stage and survival, and knockdown affected RCC cell behavior [102]. The same study generated an 18-gene prognostic model but did not identify a pRCC-specific NAT10 target mRNA. A pan-cancer analysis linked higher THUMPD1 expression with favorable survival and immune features in KIRC [103]. It did not establish THUMPD1 as an adaptor for ac^4^C in mRNA or validate the association in an RCC immunotherapy cohort.

NAT10 perturbation has also been linked to NFE2L1/GPX4 signaling and ferroptosis in cultured ccRCC cells [104]. Rescue experiments supported the functional pathway, but NFE2L1 and GPX4 mRNA ac^4^C was not measured and in vivo validation was not reported.

### 7.3. Tumor Microenvironment and Computational Studies

Current immune-related ac^4^C findings are based mainly on expression correlations and computational models. A pan-cancer NAT10 analysis reported expression and prognostic associations in renal cancer datasets but did not establish an RCC-specific immune mechanism [105]. In the pRCC study, immune-cell features were inferred computationally [102]. Its immunotherapy analysis used IMvigor210, a urothelial-cancer cohort, rather than a pRCC treatment cohort. These results do not show that ac^4^C remodels the RCC immune microenvironment or predicts response to PD-1 blockade.

### 7.4. Current Understanding and Clinical Relevance

RCC research on ac^4^C is centered on the NAT10-ANKZF1 and NAT10-NFE2L3 pathways. Remodelin reduced RNA ac^4^C and ccRCC xenograft growth in the NFE2L3 study [101]. It should be regarded as an experimental NAT10-related compound, not a specific or clinically validated inhibitor. The pRCC model, THUMPD1 associations, and pan-cancer NAT10 analyses remain retrospective [102,103,105]. No ac^4^C-derived biomarker or targeted therapy is ready for routine RCC care.

The transcript-specific NAT10 pathways and the less direct functional or computational ac^4^C studies are compared in Table 4.

## 8. Shared Pathways and Possible Crosstalk

### 8.1. Oncogenic and Metabolic Pathways

PI3K/AKT/mTOR signaling is a recurrent endpoint of several independent mRNA-modification pathways. METTL14-ITGB4 and VHL-METTL3/METTL14-PIK3R3 signaling suppressed PI3K/AKT activity [44,45]. NOP2-mediated stabilization of APOL1 mRNA activated PI3K/AKT signaling [80]. NAT10-dependent stabilization of NFE2L3 mRNA engaged the LASP1-AKT/GSK3β/β-catenin pathway [101]. These pathways reach related signaling nodes through different transcripts and can produce opposing phenotypes.

Metabolism and Hippo/YAP signaling provide a second area of overlap. m^6^A studies linked OGDHL to lipid synthesis, DBT to Hippo signaling, and inhibition of ALKBH5 and FTO to serine dependence [58,59,60]. NSUN5-dependent stabilization of ENO3 mRNA supported glycolysis [81]. NAT10-ANKZF1 promoted YAP1 nuclear localization and lymphangiogenic transcription [100]. Together, these studies suggest that different RNA modifications may independently influence common metabolic and signaling programs. Additive or opposing effects remain a testable possibility, not an established RCC mechanism.

### 8.2. Immune Regulation and Treatment Resistance

Links with the immune microenvironment are based largely on retrospective analyses. m^6^A, m^5^C, m^7^G, and ac^4^C regulator scores have been associated with immune-cell estimates, stromal content, checkpoint-gene expression, and survival [71,83,84,96,98,99,102,103,105]. The scores use different regulator sets and analytical methods, which limits direct comparison. Their shared features may reflect tumor purity, cellular composition, or broader transcriptional states. Direct mapping of these modifications in RCC immune cells remains uncommon.

Treatment resistance includes both experimental mechanisms and computational associations. YTHDC1–ANXA1 and METTL3–TRIM37 were linked to sunitinib response in RCC models [68,69], whereas the METTL3–NCAPH axis was associated with anti-PD-1 response in an immunocompetent RCC model [75]. ALYREF-CCNA1 was linked to pazopanib resistance [82]. By comparison, most m^7^G drug-sensitivity findings were computational [96,98], and ac^4^C immunotherapy associations came from retrospective or non-RCC datasets [102,103,105]. These studies identify treatment adaptation as a shared research theme, but not a coordinated cross-modification network.

### 8.3. Current Evidence for Direct Crosstalk

Three forms of overlap are relevant. Pathway convergence occurs when separate modification-target pathways affect the same signaling or phenotypic endpoint. Computational co-occurrence refers to regulator sets or inferred biological features that appear together in retrospective data. Direct molecular crosstalk would require one modification system to alter another, or two modifications to jointly control the same protein-coding mRNA. RCC studies currently document the first two forms.

MARS combined genes assigned to m^6^A, m^5^C, m^1^A, and m^7^G pathways, while another score combined m^6^A- and m^5^C-related regulators [86,87]. Such co-inclusion can generate hypotheses but does not demonstrate biochemical interaction. No included RCC study showed synergistic, competitive, or sequential regulation of one protein-coding mRNA by two modification systems. Testing this possibility will require paired measurement of both modifications, bidirectional perturbation, a shared mRNA substrate, and functional rescue in an RCC model.

## 9. Clinical Relevance in Contemporary RCC Treatment

### 9.1. Current Systemic Treatment Context

First-line treatment for metastatic ccRCC now generally uses an ICI-based combination. Options include nivolumab plus ipilimumab and ICI–TKI regimens such as pembrolizumab plus axitinib, nivolumab plus cabozantinib, and pembrolizumab plus lenvatinib [106,107,108,109,110]. Choice of regimen depends on IMDC risk, tumor histology, comorbidities, and tolerability. Sunitinib monotherapy now has a narrower role, mainly for patients who cannot receive an ICI-containing regimen [106]. The sunitinib-resistance pathways discussed above remain biologically informative, but their relevance to current combination therapy is unknown.

Treatment development continues beyond established doublets. Cabozantinib with nivolumab and ipilimumab has been evaluated in intermediate- or poor-risk disease but is not a universal standard [111]. Pembrolizumab plus lenvatinib showed activity in non-clear-cell RCC in the single-arm phase II KEYNOTE-B61 study [112]. The lack of a randomized comparator limits comparisons with other regimens.

Treatment after progression on an ICI-containing regimen presents a separate challenge. CONTACT-03 found no benefit from adding atezolizumab to cabozantinib after prior ICI exposure [113]. Belzutifan has randomized phase III data as a later-line option [114], but does not target RNA modification. These results caution against extrapolating experimental RNA-modification pathways to current treatment sequences.

### 9.2. Biomarker Potential

No RNA-modification biomarker is currently used to guide routine RCC treatment. Transcript-specific assays and rescue experiments can define biological mechanisms, but clinical utility requires a different level of validation. Most proposed signatures were derived from TCGA, GEO, pharmacogenomic estimates, or non-RCC treatment cohorts. Useful biomarkers will require prespecified thresholds, defined tissue sources, independent treatment cohorts, and prospective assessment. Current RCC guidelines base treatment choice on clinical and disease characteristics rather than RNA-modification signatures [106].

Biomarker analyses from the randomized CLEAR trial show the value of treatment-specific clinical cohorts but did not validate an RNA-modification marker [115]. RNA signatures may also differ between primary tumors and metastatic samples [116]. Future studies should therefore specify both the treatment setting and the tissue source before assessing predictive performance.

### 9.3. Therapeutic Targeting

RNA-modification-directed treatment remains experimental. FB23-2, erianin, and Remodelin have been tested only in RCC cell or animal models [61,70,101]. Remodelin is best regarded as an experimental NAT10-related compound rather than a specific, clinically validated NAT10/ac^4^C inhibitor. STC-15, an oral METTL3 inhibitor, completed a phase I study in patients with advanced malignancies, but peer-reviewed RCC-specific results are unavailable [117]. Approaches targeting m^5^C, m^7^G, or ac^4^C regulators are also largely preclinical. No RNA-modification-targeted agent is included in current RCC guidelines [106].

## 10. Methodological Considerations and Unmet Clinical Needs

Progress in RCC epitranscriptomics is constrained by assay resolution, model selection, uneven representation of RCC subtypes, and limited prospective clinical validation.

### 10.1. Detection and Site Validation

RNA-modification measurements answer different questions at different levels. Regulator expression and global abundance do not identify a modified transcript. Transcript enrichment narrows the candidate, while nucleotide-resolved assays can test a specific site. Few RCC studies combine orthogonal detection, regulator perturbation, site mutation, and functional rescue. Method performance also varies by modification and platform [118,119,120]. For m^5^C, bisulfite conversion is sensitive to incomplete conversion and RNA structure, while newer deaminase- and reader-assisted methods still require confirmation of important sites [121].

Site-level ac^4^C methods include chemical reduction and RedaC:T-seq, although analytical choices continue to affect conclusions about human mRNA ac^4^C [41,122,123]. Nanopore models can infer ac^4^C and m^5^C simultaneously, but current performance depends on training constructs and non-RCC datasets [124]. For m^6^A, GLORI provides absolute quantitative profiles at single-base resolution, while eCLIP-based methods have different sources of bias [125,126,127]. A targeted mass-spectrometry assay can distinguish m^6^A from m^6^Am, but it has not been applied to RCC samples [128]. Sample quality, input requirements, batch effects, and analytical thresholds remain practical barriers to clinical use.

### 10.2. Models, Cohorts, and RCC Subtypes

Most mechanistic work has used ccRCC cell lines, xenografts, or selected patient samples. pRCC is less well represented, and direct mRNA studies in other subtypes are scarce. It remains unclear which pathways are subtype-specific and which are shared. Retrospective results from TCGA, GEO, and pharmacogenomic resources depend on cohort composition, regulator selection, and inferred immune or drug-response phenotypes. External retrospective validation alone does not establish clinical utility. Biomarker studies need prespecified models, defined sampling sites, independent treatment cohorts, and prospective assessment [116]. KEYNOTE-B61 broadens the treatment literature for non-clear-cell RCC but does not address the lack of subtype-specific RNA-modification mechanisms [112].

### 10.3. Translational Gaps

The main translational gap is the distance between experimental mechanisms and treatment-specific clinical testing. Several m^6^A and m^5^C pathways influence sunitinib or pazopanib response in preclinical models, yet none has been tested in ICI–ICI or ICI–TKI resistance models. Immune conclusions often rely on regulator expression, deconvolution algorithms, or non-RCC treatment cohorts rather than direct mapping in RCC immune cells. Proposed biomarkers are not used in routine treatment, and current guidelines include no RNA-modification-targeted agent [106]. Clinical studies will need a defined assay or intervention, an appropriate treatment setting, and prospectively collected RCC cohorts.

### 10.4. Future Research Priorities

Other mRNA modifications remain understudied in RCC. m^1^A has been detected in mRNA, but its abundance, distribution, and regulatory machinery are less clearly resolved than those of m^6^A [129,130]. Two recent ccRCC studies used TCGA-KIRC together with GEO or ArrayExpress cohorts to relate m^1^A-related regulator expression patterns to survival and computationally inferred immune features [131,132]. A separate multi-modification analysis that included m^1^A-related regulators identified ccRCC subtypes with different clinical and immune characteristics [133]. None of these studies directly measured m^1^A on a defined protein-coding mRNA. They therefore support retrospective associations rather than an m^1^A-dependent mechanism or a clinically validated biomarker. Current RCC data remain insufficient for a separate mechanistic section. Priorities include nucleotide-resolved mapping with orthogonal confirmation, better representation of pRCC and other subtypes, and paired samples collected before treatment and after resistance. Models should reproduce current ICI–TKI or ICI–ICI exposure. Multi-omics analyses can nominate candidates, but direct site validation and clinical testing remain necessary.

## 11. Conclusions

mRNA modifications contribute to several aspects of RCC biology, but the depth of support differs across modification classes. m^6^A has the most extensive transcript-level literature and links defined regulator–mRNA pathways to tumor growth, metabolic adaptation, immune phenotypes, and TKI resistance. m^5^C and ac^4^C each have a smaller number of direct mRNA mechanisms. Current m^7^G studies mainly describe regulator expression, functional perturbation, and retrospective signatures, without nucleotide-resolved validation of an internal RCC mRNA site.

Separate modification pathways converge on PI3K/AKT, Hippo/YAP, metabolism, immune regulation, and treatment resistance. Direct molecular crosstalk has not yet been demonstrated in RCC. RNA-modification biomarkers and targeted agents are also not part of routine care. The field therefore offers useful mechanistic questions but remains clinically immature. Progress will depend on site-resolved experiments, broader RCC subtype coverage, models that reflect current treatment, and prospective clinical validation.

## Figures and Tables

**Table 1:** RCC studies involving m^6^A modification or m^6^A-related regulators.

Modification	RNA Species	Regulator–Target Axis	RCC Subtype	Study Design/Data Source	Model/Cohort	Direct Modification Assay	Functional/Clinical Validation	Evidence Category	Permitted Conclusion/Clinical Status	Ref.
m^6^A	TGF-β/SMAD-family mRNAs (site evidence for SMAD4)	IGF2BP2–TGF-β/SMAD mRNAs	ccRCC	Mechanistic	TCGA, tissue arrays, paired specimens, cells and xenografts	MeRIP/RIP; SELECT and mutant reporters for SMAD4 sites	Stability, pull-down, rescue and xenografts	C1a	Context-dependent growth suppression and metastasis promotion; strongest site evidence for SMAD4	[55]
m^6^A	KLK4 mRNA	METTL16–IGF2BP1–KLK4	ccRCC	Mechanistic	Cells and animal models	KLK4 MeRIP-PCR and candidate-site reporter	METTL16/KLK4 perturbation and rescue	C1b	Preclinical transcript-level mechanism; no single-base quantification	[48]
m^6^A	ZFP14 mRNA	METTL14–IGF2BP2–ZFP14–STAT3	ccRCC	Mechanistic	Cells, 50 paired samples and mouse models	MeRIP-seq/qPCR and candidate-site mutant reporters	Perturbation, stability, rescue and in vivo validation	C1b	Preclinical tumor-suppressive mechanism; no chemical single-base assay	[50]
m^6^A	POLQ mRNA	FTO–POLQ	ccRCC	Mechanistic	Cells, specimens and public data	MeRIP-seq/qPCR and mutant reporter	Perturbation, stability and rescue	C1b	Preclinical proliferation/genome-stability mechanism	[51]
m^6^A	Netrin-4 mRNA	IGF2BP2–Netrin-4	ccRCC	Mechanistic	43 patients, controls and RCC cells; no in vivo model	Netrin-4 MeRIP-qPCR, RIP and candidate-site reporter	Perturbation, stability and knockdown rescue	C1b	Preclinical migration/invasion mechanism; not a validated clinical biomarker	[56]
m^6^A	ACLY mRNA	CDK13–METTL16–YTHDC2–ACLY	ccRCC	Mechanistic/metabolic	Cells, specimens and xenografts	ACLY MeRIP-qPCR; candidate-site mutant reporters	Perturbation, stability, reporters, metabolism and rescue	C1b	Preclinical lipogenic mechanism; candidate rather than chemically resolved sites	[62]
m^6^A	HMOX1 mRNA	Loganin–IGF2BP2–HMOX1	RCC	Mechanistic/preclinical compound	RCC cells and xenografts	HMOX1 MeRIP-qPCR; candidate-site reporter; no chemical nucleotide resolution	IGF2BP2 rescue, stability, ferroptosis and xenograft assays	C1b	Preclinical ferroptosis mechanism; Loganin not clinically validated	[66]
m^6^A	MANF mRNA	METTL3–IGF2BP1–MANF	RCC	Mechanistic/stress response	Cells, specimens and related models	MANF MeRIP-qPCR and candidate-site mutant reporter	Perturbation, stability and MANF rescue	C1b	Preclinical ER-stress-tolerance mechanism	[67]
m^6^A	sFRP4 mRNA	ZC3H13–sFRP4–Wnt/β-catenin	RCC	Mechanistic resistance	Small clinical series, resistant cells and xenografts	sFRP4 MeRIP-qPCR; predicted-site reporter	ZC3H13/sFRP4 rescue, stability, pathway and in vivo assays	C1b	Preclinical sunitinib-resistance mechanism; limited cohort/model size	[76]
m^6^A	ITGB4 mRNA	METTL14–ITGB4	ccRCC	Mechanistic	Cells, specimens, xenograft	Transcript-level m^6^A enrichment; no nucleotide-level site	Perturbation, stability, phenotype, in vivo	C1c	Preclinical direct transcript mechanism	[44]
m^6^A	PIK3R3 mRNA	VHL–METTL3/METTL14–PIK3R3	ccRCC	Mechanistic/multi-omics	RCC models and specimens	Transcript-level m^6^A evidence; no nucleotide-level site	Genetic perturbation, stability, pathway and phenotype	C1c	Preclinical VHL-linked mechanism	[45]
m^6^A	MYC mRNA	VIRMA/KIAA1429–MYC	ccRCC	Mechanistic	Cells, specimens/public data, xenograft	m^6^A enrichment and RNA binding; no nucleotide-level site	Knockdown, stability, rescue, in vivo	C1c	Preclinical transcript mechanism	[46]
m^6^A	ZNF677 mRNA	METTL3–IGF2BP2/YTHDF1–ZNF677	RCC	Mechanistic	Cells, specimens, xenograft	Transcript-level m^6^A enrichment	Stability/translation, rescue, in vivo	C1c	Preclinical transcript mechanism	[47]
m^6^A	PLOD2 mRNA	Hypoxia–METTL3–PLOD2	RCC	Mechanistic/hypoxia	Cells, TCGA, tissues and mouse models	PLOD2 MeRIP-RT-qPCR; no nucleotide-level site	Catalytic mutant, perturbation, rescue and in vivo validation	C1c	Transcript-level hypoxia mechanism; not site-specific	[52]
m^6^A	ZHX2 mRNA	METTL3–IGF2BP1–ZHX2	RCC	Mechanistic	43 RCC/43 control tissues, cells and xenografts	ZHX2 MeRIP-qPCR and RIP; sites predicted only	Perturbation, stability, rescue and xenografts	C1c	Preclinical stability mechanism; no nucleotide-level validation	[53]
m^6^A	BCL2 and E2F2 mRNAs	YTHDC1 K82 lactylation–BCL2/E2F2	RCC	Mechanistic/reader regulation	120 paired samples, cells and mouse models	Target MeRIP-qPCR and YTHDC1 RIP/CLIP; no m^6^A nucleotide site	K82 mutants, reader mechanism, rescue and in vivo validation	C1c	Protein lactylation modulates reader function; not dual-RNA-modification crosstalk	[54]
m^6^A	ANLN mRNA	CAF/lactate–RBM15B–ANLN	pRCC	Mechanistic/stromal	16 paired tissues, RCC cells and fibroblast/CAF models	ANLN MeRIP-qPCR and RBM15B RIP; no nucleotide-level site	RBM15B knockdown and ANLN rescue; cell phenotypes	C1c	Stromal m^6^A mechanism; protein lactylation is not RNA-modification crosstalk	[57]
m^6^A	OGDHL mRNA	FTO–OGDHL	ccRCC	Mechanistic	Cells, specimens, xenograft	Transcript-level m^6^A enrichment	Stability, metabolic and phenotypic validation	C1c	Preclinical metabolic mechanism	[58]
m^6^A	DBT mRNA	METTL3–DBT	ccRCC	Mechanistic	Cells, specimens, xenograft	Transcript-level m^6^A enrichment	Stability, pathway, rescue, in vivo	C1c	Preclinical metabolic mechanism	[59]
m^6^A	PSAT1 and serine-pathway mRNAs	L-2HG–ALKBH5/FTO–m^6^A	Kidney cancer (not subtype-specific)	Mechanistic/multi-omics	Cell and animal models	m^6^A profiling with target validation	Metabolic perturbation and rescue	C1c	Preclinical metabolic vulnerability; not an RCC biomarker	[60]
m^6^A	SIK2 mRNA	FTO–IGF2BP2–SIK2	ccRCC	Mechanistic/drug	Cells, specimens, xenograft/PDX	Transcript-level m^6^A and reader binding	Stability, autophagy, rescue; FB23-2 in PDX	C1c	Preclinical mechanism and intervention	[61]
m^6^A	Poly(A)+ mRNA/global peaks	FH loss/fumarate–mRNA m^6^A	FH-deficient RCC (HLRCC model)	Mechanistic/omics	Isogenic patient-derived FH-deficient/restored cells	LC-MS/MS, m^6^A-IP-seq/qPCR; limited SELECT in main article	FH restoration and stability/translation profiling	C1c	Global/peak rewiring; no proof that hypermethylation caused global stability or translation change	[65]
m^6^A	ANXA1 mRNA	YTHDC1–ANXA1	ccRCC	Mechanistic resistance	Cells, specimens, xenograft	m^6^A-marked transcript and reader binding; no site validation	Stability, MAPK signaling, sunitinib sensitivity	C1c	Preclinical sunitinib-resistance mechanism	[68]
m^6^A	TRIM37 mRNA	METTL3–TRIM37	RCC	Mechanistic resistance	Cells, tissues, animal models	Transcript-level m^6^A enrichment; no nucleotide-level site	Stability, rescue, sunitinib resistance	C1c	Preclinical resistance mechanism	[69]
m^6^A	ALOX12 and TP53 mRNAs	Erianin–m^6^A–ALOX12/TP53	RCC (stem-cell models)	Preclinical compound	Cell and animal models	Transcript-level m^6^A signals; no site validation	Ferroptosis and phenotype	C1c	Experimental compound evidence only	[70]
m^6^A	UNC5CL mRNA	ZC3H13–YTHDC1–UNC5CL–CSF2	RCC	Mechanistic/immune	Cells, specimens, xenografts and MDSC assays	MeRIP-seq and UNC5CL MeRIP-qPCR; no nucleotide-level site	Perturbation, stability, NF-κB inhibition and in vivo rescue	C1c	Preclinical immune-microenvironment mechanism; not an ICI biomarker	[72]
m^6^A	NCAPH mRNA	METTL3–YTHDC1/IGF2BP3–NCAPH	ccRCC	Mechanistic and immune-response study	ccRCC cells and specimens, xenografts, T-cell coculture, and an immunocompetent mouse model	Transcript-level m6A enrichment and METTL3 perturbation; no nucleotide-level site validation	Nuclear export, mRNA stability, T-cell killing, PD-L1/β-catenin signaling, and anti-PD-1 mouse experiment	C1c	Preclinical m6A-centered immune-evasion and anti-PD-1 response mechanism; no clinical validation	[75]
m^6^A	SYNM mRNA	YTHDF3–SYNM	ccRCC	Mechanistic resistance	NVP-BEZ235-resistant ccRCC model	m^6^A-RIP-seq, MeRIP-qPCR and YTHDF3 RIP	YTHDF3/SYNM perturbation and drug-sensitivity restoration	C1c	Experimental resistance to nonstandard NVP-BEZ235; no clinical validation	[77]
m^6^A	TMSB10 mRNA	KIAA1429–TMSB10	ccRCC	Mechanistic (abstract only)	Samples, cells and xenografts reported in abstract	Abstract reports TMSB10 MeRIP; site resolution not assessable	Abstract reports perturbation, rescue and xenografts	C1d	Provisional transcript-level mechanism; not site-specific or clinically validated	[49]
m^6^A	Transcriptome-wide candidate mRNAs	Nanopore direct-RNA m^6^A landscape	ccRCC	Exploratory method/omics	One paired tumor-adjacent sample plus public datasets	Algorithmic Nanopore calling; no orthogonal site validation	No mechanistic perturbation or rescue	C1d	Feasibility evidence only; not a mechanism or biomarker	[78]
m^6^A-related	ACSM3 mRNA	METTL14–ACSM3–GATA5/HMGCS2	ccRCC	Regulator–target mechanism	30 paired tissues, TCGA, cells and xenografts	Prediction and mutant reporters; no target-mRNA MeRIP in audited main text	Perturbation, metabolic assays and xenografts	C2	Regulator–target mechanism without direct ACSM3 m^6^A measurement	[63]
m^6^A-related	SLC1A5 mRNA	FTO–SLC1A5	VHL-deficient ccRCC	Regulator–target/metabolic	Cells, xenografts and PDX	No new target-transcript assay; modification link relies on prior evidence	FTO/SLC1A5 perturbation, metabolic tracing and rescue	C2	Preclinical metabolic mechanism; direct m^6^A evidence not newly established	[64]
m^6^A-related	IL15RA mRNA	STAT1/METTL3–IL15RA–ZEB1/NF-κB	ccRCC	Regulator–target mechanism	Public data, cells and specimens	No direct IL15RA modification assay	Perturbation, stability and pathway experiments	C2	Regulator–target mechanism; not a direct site or ICI biomarker	[73]
m^6^A-related	HSPA13 mRNA	YTHDF3–HSPA13	ccRCC	Regulator–target/immune	Cells, public cohorts and mouse immune models	YTHDF3–HSPA13 RIP; HSPA13 m^6^A inferred only	Phase-separation mutants, stability, mechanism and rescue	C2	Regulator–target immune-evasion mechanism; not a direct site or ICI biomarker	[74]
m^6^A-related	No defined mRNA substrate	m^6^Ascore	ccRCC	Retrospective computational	Public RCC cohorts and one anti-PD-1 cohort	None	Retrospective survival and immune associations	C4	Exploratory; not a validated predictive biomarker	[71]

Note: Each row represents one original RCC study. C1a, site-directed or orthogonally supported modification-dependent evidence; C1b, candidate-site functional evidence without chemical single-nucleotide confirmation; C1c, transcript- or peak-level modification evidence without site validation; C1d, global, method-dependent, non-orthogonally validated, or abstract-reported mapping evidence; C2, regulator–target mRNA mechanism without direct measurement of the modification on that transcript; C4, retrospective computational evidence. C1a–C1d describe assay resolution and do not represent risk-of-bias or clinical-maturity grades. Clinical interpretations remain limited by validation design and treatment setting. Abbreviations: CAF, cancer-associated fibroblast; ccRCC, clear cell renal cell carcinoma; CLIP, cross-linking and immunoprecipitation; HLRCC, hereditary leiomyomatosis and renal cell carcinoma; ICI, immune checkpoint inhibitor; LC–MS/MS, liquid chromatography–tandem mass spectrometry; MDSC, myeloid-derived suppressor cell; MeRIP, methylated RNA immunoprecipitation; PDX, patient-derived xenograft; pRCC, papillary renal cell carcinoma; RCC, renal cell carcinoma; RIP, RNA immunoprecipitation; SELECT, single-base elongation- and ligation-based qPCR amplification method; TCGA, The Cancer Genome Atlas.

**Table 2:** RCC studies involving m^5^C modification, m^5^C-related regulators, or mixed-modification signatures.

Modification	RNA Species	Regulator–Target Axis	RCC Subtype	Study Design/Data Source	Model/Cohort	Direct Modification Assay	Functional/Clinical Validation	Evidence Category	Permitted Conclusion/Clinical Status	Ref.
m^5^C	PEBP1 mRNA	PEBP1P2–YBX1/ELAVL1–PEBP1	ccRCC	Mechanistic	Cells, specimens, animal models	Transcript m^5^C and targeted demethylation	Stability, rescue, metastatic phenotypes	C1a	Direct mRNA-centered mechanism; pseudogene is upstream	[79]
m^5^C	APOL1 mRNA	NOP2–YBX1–APOL1	ccRCC	Mechanistic	Cells, specimens, xenograft	Bisulfite sequencing, MeRIP/RIP and site-mutant reporters	Stability, rescue, pathway, in vivo	C1a	Site-directed preclinical mechanism	[80]
m^5^C	CCNA1 mRNA	ALYREF–CCNA1	RCC	Mechanistic resistance	Resistant cells and mouse models	Anti-m^5^C/ALYREF RIP and predicted-site mutant reporters	Reader mutant, stability, rescue, in vivo	C1b	Preclinical pazopanib-resistance mechanism	[82]
m^5^C	ENO3 mRNA	NSUN5–ENO3	ccRCC	Mechanistic	Cells, specimens, xenograft	Transcript m^5^C enrichment; no nucleotide-level site	Stability, glycolysis, in vivo	C1c	Preclinical transcript mechanism	[81]
m^5^C-related	No validated target mRNA	Regulator-expression panel	ccRCC	Retrospective with limited cell assays	Public datasets and cell lines	None	Expression correlations and limited function	C3	Candidate regulators only	[89]
m^5^C-related	No validated target mRNA	NSUN6 expression/function	ccRCC	Regulator expression/function	TCGA/GEO, tissues and ccRCC cells	None	Expression and cell proliferation/migration/invasion assays	C3	Regulator-level evidence only; no m^5^C-modified mRNA substrate or clinical tool	[90]
m^5^C-related	No validated target mRNA	NSUN5 expression/function	ccRCC	Regulator expression/function	TCGA, SYSUCC cohort and ccRCC cells	None	Expression, perturbation and exploratory prognosis	C3	Regulator-level evidence only; no specific axis involving m^5^C in mRNA	[91]
m^5^C-related	No validated target mRNA	NSUN5–p53-pathway phenotype	ccRCC	Regulator function	TCGA, ccRCC cells and xenografts	None	NSUN5 knockdown, cell phenotypes and xenografts	C3	Functional evidence only; no specific mechanism involving m^5^C in mRNA	[92]
m^5^C-related	LDHA mRNA	YBX1–LDHA–NF-κB	ccRCC	Regulator–target/function study	63 RCC specimens, ccRCC cells, and orthotopic xenografts	None	Expression, transcription/protein interaction, LDHA inhibition/rescue, and in vivo validation	C3	Functional YBX1–LDHA axis without demonstrated m^5^C dependence	[93]
m^5^C-related	No defined mRNA substrate	m^5^C patterns/score	ccRCC	Retrospective multi-cohort	Public datasets	None	Computational immune and survival associations	C4	Exploratory association only	[83]
m^5^C-related	No defined mRNA substrate	M5CRMRGI	ccRCC	Retrospective multi-omics	Public bulk, single-cell and spatial datasets	None	Computational immune and drug-response inference	C4	Not validated for ICI or mTOR-inhibitor benefit	[84]
m^5^C-related	Regulator signature	ALYREF/DNMT3B/YBX1 signature	pRCC	Retrospective computational	TCGA-KIRP, ICGC and connectivity map	None	External retrospective validation and drug inference	C4	Exploratory; not treatment-guiding	[85]
m^5^C-related	No validated target mRNA	m^5^C-regulator patterns/m^5^Cscore	ccRCC	Retrospective computational	TCGA/ICGC public cohorts	None	Subtype, immune-infiltration and drug-sensitivity inference	C4	Exploratory associations; not a treatment predictor	[88]
m^6^A/m^5^C-related	Five-gene score	NOP2/METTL14/NSUN5/HNRNPA2B1/ZC3H13	ccRCC	Retrospective computational	Public datasets	None	Survival and immune associations	C4	Mixed-modification exploratory score	[87]
Multi-modification-related	Gene signature	MARS	ccRCC	Retrospective computational	TCGA, GEO, and ArrayExpress cohorts	None	Retrospective survival, immune, and drug-response inference	C4	Mixed-modification exploratory score; not m^5^C-specific or clinically validated	[86]

Note: C3, regulator expression or function without a verified modified target mRNA. Abbreviations: GEO, Gene Expression Omnibus; ICGC, International Cancer Genome Consortium; SYSUCC, Sun Yat-sen University Cancer Center; TCGA-KIRP, The Cancer Genome Atlas Kidney Renal Papillary Cell Carcinoma project.

**Table 3:** RCC studies involving m^7^G-related regulators and computational signatures.

Modification	RNA Species	Regulator–Target Axis	RCC Subtype	Study Design/Data Source	Model/Cohort	Direct Modification Assay	Functional/Clinical Validation	Evidence Category	Permitted Conclusion/Clinical Status	Ref.
m^7^G-related	No validated modified RCC mRNA	METTL1 expression/signature	ccRCC	Retrospective with IHC	TCGA and tissue microarray	None	Expression/IHC and computational model	C3; C4 component	Regulator association; not proof of internal m^7^G in mRNA	[94]
m^7^G-related	Candidate mRNAs inferred partly from non-RCC data	METTL1/BUD23	ccRCC	Regulator-function study	ccRCC cell and 3D models; non-RCC m^7^G-data reanalysis	No RCC nucleotide-resolved site validation	Knockdown and functional phenotypes	C3	Regulator function only; internal sites unproven	[95]
m^7^G-related	No validated modified RCC mRNA	WDR4/regulator subtypes	ccRCC	Retrospective with cell perturbation	Public data and RCC cell lines	None	WDR4 knockdown, experimental sorafenib/sunitinib IC50 assays, and additional computational drug-sensitivity analyses	C3; C4 component	Regulator-level function and experimental cell-line drug response; no validated internal m^7^G site in mRNA or clinical prediction	[96]
m^7^G-related	Five-gene signature	CYFIP2/EIF4A1/NUDT1/NUDT10/NUDT4	ccRCC	Retrospective computational	TCGA and E-MTAB-1980	None	External retrospective survival association	C4	Not a clinically validated biomarker	[97]
m^7^G-related	Regulator patterns/signature	m^7^G patterns/score	ccRCC	Retrospective multi-omics	Public cohorts	None	Computational immune and drug associations	C4	Exploratory association only	[98]
m^7^G-related	Regulator patterns	m^7^G/immune patterns	ccRCC	Retrospective computational	Public cohorts	None	Computational immune associations	C4	Exploratory association only	[99]

Note: “C3; C4 component” indicates that a study contains both regulator-level functional evidence and a retrospective computational component. Abbreviations: 3D, three-dimensional; IC50, half-maximal inhibitory concentration; IHC, immunohistochemistry.

**Table 4:** RCC studies involving ac^4^C modification or ac^4^C-related regulators.

Modification	RNA Species	Regulator–Target Axis	RCC Subtype	Study Design/Data Source	Model/Cohort	Direct Modification Assay	Functional/Clinical Validation	Evidence Category	Permitted Conclusion/Clinical Status	Ref.
ac^4^C	ANKZF1 mRNA	NAT10–ANKZF1–YAP1	ccRCC	Mechanistic	Cells, tissues, xenograft	ac^4^C detection with site-specific mutation	Rescue, in vivo and lymphangiogenesis	C1a	Site-directed preclinical mechanism	[100]
ac^4^C	NFE2L3 mRNA	HIF-1α–NAT10–NFE2L3–LASP1	ccRCC	Mechanistic/drug	Cells, tissues and xenograft	acRIP-seq/qPCR, RIP and mutant reporter	Stability, rescue, in vivo; Remodelin	C1b	Preclinical mechanism and intervention	[101]
ac^4^C-related	No validated pRCC target mRNA	NAT10 expression/18-gene model	pRCC	Retrospective with limited function	Public/single-cell data, RCC cells, non-RCC ICI cohort	None	NAT10 knockdown and computational model	C3; C4 component	No pRCC ac^4^C axis or ICI prediction	[102]
ac^4^C-related	No validated target mRNA	NAT10–NFE2L1/GPX4	ccRCC	Regulator function/ferroptosis	ccRCC cells, 30 paired tissues and GEO	No NFE2L1 or GPX4 mRNA ac^4^C assay	NAT10 perturbation and NFE2L1/GPX4 rescue; no in vivo validation	C3	Functional ferroptosis axis; not a direct mechanism involving ac^4^C in mRNA	[104]
ac^4^C-related	No validated target mRNA	THUMPD1 expression	ccRCC	Retrospective pan-cancer	Public datasets (including TCGA-KIRC) and non-RCC immunotherapy cohorts	None	Expression and immune associations only	C4	Not an adaptor for ac^4^C in mRNA or RCC ICI biomarker	[103]
ac^4^C-related	No validated target mRNA	NAT10 expression	RCC	Retrospective pan-cancer	Public datasets	None	Expression and immune associations	C4	No RCC-specific immune mechanism	[105]

Abbreviations: acRIP, ac4C RNA immunoprecipitation; TCGA-KIRC, The Cancer Genome Atlas Kidney Renal Clear Cell Carcinoma project.

## Data Availability

No new data were created or analyzed in this study. Data sharing is not applicable to this article.
